# Structure of Active Sites of Fe-N-C Nano-Catalysts for Alkaline Exchange Membrane Fuel Cells

**DOI:** 10.3390/nano8120965

**Published:** 2018-11-22

**Authors:** Hirofumi Kishi, Tomokazu Sakamoto, Koichiro Asazawa, Susumu Yamaguchi, Takeshi Kato, Barr Zulevi, Alexey Serov, Kateryna Artyushkova, Plamen Atanassov, Daiju Matsumura, Kazuhisa Tamura, Yasuo Nishihata, Hirohisa Tanaka

**Affiliations:** 1 Advanced R&D Department, Daihatsu Motor Co. Ltd., 3000 Yamanoue, Ryuo, Gamo, Shiga 520-2593, Japan; Hirofumi_Kishi@dk.daihatsu.co.jp (H.K.); Tomokazu_Sakamoto@dk.daihatsu.co.jp (T.S.); Susumu_Yamaguchi@dk.daihatsu.co.jp (S.Y.); Takeshi_Katou@dk.daihatsu.co.jp (T.K.); 2 Pajarito Powder Limited Liability Company (LLC), 3600 Osuna Rd NE, Suite 309, Albuquerque, NM 87102, USA; bhalevi@pajaritopowder.com (B.Z.); aserov@pajaritopowder.com (A.S.); 3 Department of Chemical & Biological Engineering, Center for Micro-Engineered Materials (CMEM), University of New Mexico, Albuquerque, NM 87131, USA; kartyush@unm.edu (K.A.); plamen@unm.edu (P.A.); 4 Quantum Beam Science Center, Japan Atomic Energy Agency, 1-1-1, Koto, Sayo, Hyogo 679-5148, Japan; daiju@spring8.or.jp (D.M.); tkazu@spring8.or.jp (K.T.); yasuon@spring8.or.jp (Y.N.); 5 Department of Nanotechnology for Sustainable Energy, School of Science and Technology, Kwansei Gakuin University, 2-1 Gakuen, Sanda, Hyogo 669-1337, Japan; hirohisa.tanaka@kwansei.ac.jp

**Keywords:** anion exchange membrane fuel cells (AEMFCs), iron-nitrogen-carbon electrocatalysts (Fe-N-C), HO_2_^−^ generation, oxygen reduction reaction

## Abstract

Platinum group metal-free (PGM-free) catalysts based on transition metal-nitrogen-carbon nanomaterials have been studied by a combination of ex situ and in situ synchrotron X-ray spectroscopy techniques; high-resolution Transmission Electron Microscope (TEM); Mößbauer spectroscopy combined with electrochemical methods and Density Functional Theory (DFT) modeling/theoretical approaches. The main objective of this study was to correlate the HO_2_^−^ generation with the chemical nature and surface availability of active sites in iron-nitrogen-carbon (Fe-N-C) catalysts derived by sacrificial support method (SSM). These nanomaterials present a carbonaceous matrix with nitrogen-doped sites and atomically dispersed and; in some cases; iron and nanoparticles embedded in the carbonaceous matrix. Fe-N-C oxygen reduction reaction electrocatalysts were synthesized by varying several synthetic parameters to obtain nanomaterials with different composition and morphology. Combining spectroscopy, microscopy and electrochemical reactivity allowed the building of structure-to-properties correlations which demonstrate the contributions of these moieties to the catalyst activity, and mechanistically assign the active sites to individual reaction steps. Associated with Fe-N_x_ motive and the presence of Fe metallic particles in the electrocatalysts showed the clear differences in the variation of composition; processing and treatment conditions of SSM. From the results of material characterization; catalytic activity and theoretical studies; Fe metallic particles (coated with carbon) are main contributors into the HO_2_^−^ generation.

## 1. Introduction

Platinum has a limited availability on Earth, and its substitution with a platinum group metal-free (PGM-free) electrocatalyst is needed in order for the fuel cell to become feasible and widely spread technology [1,2,3].

Among several classes of PGM-free catalysts, transition metal-nitrogen-carbon (M-N-C) nanomaterials, where the transition metal (M) is usually Fe, Ni, Co (or few others) are most often studied and discussed. M-N-C nanomaterials are highly active electrocatalysts for oxygen reduction reaction (ORR). An iron-nitrogen-carbon electrocatalyst (Fe-N-C) has attracted attention due to its highest ORR activity among the other M-N-C electrocatalysts and demonstrated multiple synthesis protocols to yield desired nanomaterial. Fe-N-C have been employed in experimental proton exchange membrane fuel cells (PEMFC) and have demonstrated promise, while still being much inferior to PGM catalysts [4]. In contrast, in alkaline media M-N-C demonstrate performance at par with PGM, as such find applications as cathode materials in Anion Exchange Membrane Fuel Cells (AEMFCs) [5].

In general, Fe-N-Cs are synthesized through pyrolysis of an N-containing organic precursor, serving as a source of the carbonaceous matrix and a transition metal salt as a source of the metal-containing moieties in the final nanomaterial [6]. Often, the metal is present in the precursor compound, as in metal organic frameworks (MOF) or is introduced in a specific stage of the catalyst preparation [7,8,9]. Substantial efforts have been made towards identifying the structure of the active sites in M-N-C nanomaterials. Current understanding of the M-N-C (and specifically Fe-N-C) catalyst structure involves two central hypotheses: (i) transition metal is atomically dispersed and is built into N-containing carbonaceous matrix and is the site on which di-oxygen binds prior to electro-reduction steps [10,11], and (ii) transition metal form nano-particles of ether reduced metal or metal carbide, which are immersed in carbonaceous matrix and induce ORR catalytic properties onto that matrix, without directly participating in the reaction [12]. Explicit understanding of the chemical structure of the active sites in M-N-C nanomaterials is additionally obscured by the fact that several Fe-containing and N-containing moieties are catalytically active in various individual steps of ORR [13]. This interplay between multiplicity of the chemical structure of the individual active sites and their participation in reaction mechanism, especially in the generation of intermediate HO_2_^−^, have yet to be revealed in full.

Under alkaline conditions, the kinetics of the Oxygen Reduction Reaction (ORR) on the cathode is enhanced leading to improved overall fuel cell efficiency. Anion exchange membrane fuel cells (AEMFCs) with an alkaline liquid electrolyte such as KOH (aq) are the best performing of all known conventional hydrogen oxygen fuel cell types. The application of alkaline conditions at the electrodes opens the potential to use a range of low-cost PGM-free catalysts. In order to enable liquid electrolyte-free AEMFCs, a number of groups have begun research efforts devoted to fabrication and engineering of anion-exchange membranes and ionomer solutions [14,15,16,17,18,19,20]. Also, there are incentives to develop novel materials for AEMFC systems that have the potential to alleviate or eliminate the technical issues associated with liquid electrolyte systems. This may include the use of a much more diverse selection of potential fuels that are thermodynamically favorable in alkaline media.

As one of the most promising ORR electrocatalysts for AEMFC, transition metal-nitrogen-carbon catalysts (M-N-C) have been intensively studied [21]. The performance of these catalysts is remarkably better in a direct hydrazine fuel (DHFC) cell in comparison with a polymer electrolyte fuel cell reaching a power density of 500 mW/cm^2^ [1,21,22]. Jasinski was the first to show the potential of utilizing a transition metal macrocycle compounds for alkaline oxygen electroreduction [23]. In order for the M-N-C catalysts to achieve their highest overall performance, they must have a well-developed morphology and high density of active sites [24,25,26].

For over a decade, the University of New Mexico has been developing an original synthetic method for M-N-C catalysts preparation called the Sacrificial Support Method (SSM) [4,27]. An industrial application for the SSM was successfully brought onto the catalyst market by Pajarito Powder under the trademarked name VariPore™ [28]. The SSM synthesis produces material with multiple controlled surface defects within a carbonaceous network and an internal network of connected pores with adjustable pore size distribution (PSD). The key chemical features of these electrocatalysts result in high activity and excellent stability. Additionally, it is significant that the formulations, processing and treatment conditions of SSM can be optimized for the best ORR performance at AEMFCs conditions.

To rationally design active and durable electrocatalysts for AEMFCs, the mechanism and the nature active site of ORR in alkaline conditions need to be identified. Especially, it is crucially required to reveal the mechanism of HO_2_^−^ species generation because HO_2_^−^ is a source of OH radical [29] which decreases the longevity of anion-exchange membranes and ionomer dispersions.

To reveal the mechanism of HO_2_^−^ generation, Fe-N-C ORR electrocatalysts synthesized with variation of several parameters were analyzed by synchrotron X-ray radiation to build structure-to-properties correlations on the basis of our previous works [30,31,32,33]. The structure of electrocatalysts, which is associated with Fe-N_x_ motive and the presence of Fe metallic particle showed the clear difference in the variation of composition, processing and treatment conditions of SSM. From the results of material characterization X-ray Absorption Fine Structure (XAFS), Scanning Transmission Electron Microscope (STEM), catalytic activity test (Rotating Ring Disk Electrode, RRDE) and theoretical studies (DFT calculation), it was indisputably shown that Fe metallic particle (coated with carbon) are main contributors to the HO_2_^−^ generation.

In parallel to the analysis of the mechanism of HO_2_^−^ generation, we performed Mößbauer spectroscopy measurements and in situ XAFS analysis of the iron species to derive a guideline for the design of highly active ORR catalysts.

## 2. Materials and Methods

### 2.1. Catalyst Preparation

The catalysts were synthesized by modified SSM [30,31]. Iron nitrate (2.5 g, Fe(NO_3_)_3_·9H_2_O, Sigma Aldrich) was mechanically mixed with 25 g of the nitrogen-rich organic precursors (Nicarbazin and Pipemidic acid) and 10 g of LM-150 fumed silica (Cabot Cab-O-sil^®^, surface area ~150 m^2^/g). The pre-mixed material was loaded into a 100 mL agate ball-mill jar with 16 agate balls (diameter 1 cm). The mixture was subjected to ball-milling at 450 rpm for 1 h. The homogeneous powder was pyrolyzed at T = 950 °C, t = 30 min in the flow of Ultra High Purity (UHP) Nitrogen, 100 ccm. After heat treatment, silica was removed by 20 wt. % HF (1st acid treatment), followed by washing with DI water until neutral pH was reached. The obtained powder was dried overnight at T = 85 °C. In order to remove Fe metallic particles, the catalysts were acid treated with 1 M HNO_3_ (2nd acid treatment). The abbreviations of each catalyst sample are shown in Table 1 together with the precursor and acid treatment history.

### 2.2. XAFS Data Collection and Analysis

X-ray absorption fine structure (XAFS) measurements were carried out at line BL14B2 line of SPring-8 as in our previous work [32,33]. During in-situ XAFS analysis, potential was set at 0.25 V, 0 V, −0.20 V, −0.40 V, −0.60 V, −0.75 V and −0.90 V vs. Hg/HgO (1.174 V, 0.924 V, 0.724 V, 0.524 V, 0.324 V, 0.174 V and 0.024 V vs. RHE). XAFS data processing was done using EXAFS analysis software (Ifeffit; University of Chicago, Chicago, IL, USA) for fitting.

### 2.3. Rotating Ring-Disk Electrode (RRDE) Preparation and Testing

The catalyst layer setup to the disk electrode and electrochemical measurement has been done as our previous work [32]. The ratio of HO_2_^−^ generation was calculated using Equation (1).

P(HO_2_^−^) (%) = 2 × I_r_/(N × I_d_ + I_r_) × 100  (N = 0.38 in this work)(1)

### 2.4. STEM Analysis

Scanning Transmission Electron Microscope (STEM) and STEM-EDS (JEM-ARM200F, Japan Electron Optics Laboratory Company Limited; Tokyo, Japan) with the voltage acceleration of 200 kV were performed to analyze catalyst morphology and composition.

### 2.5. HAXPES Analysis

Hard X-ray Photo Electron Spectroscopy (HAXPES) measurements were carried out at BL46XU and BL47XU of SPring-8; Hyogo, Japan. The source X-ray energy was 7940 eV. Fe2p spectra were acquired. Spectra were charge calibrated to the binding energy for Au standard plate of 84 eV (Au4f).

### 2.6. ^57^Fe Mößbauer Spectroscopy

Mößbauer measurements were made to characterize the iron compounds within each catalyst. The spectra were recorded at room temperature with a CMCA-550 (WissEl; Starnberg, Germany) equipped with a constant electronic drive system with a triangular reference waveform (Halder Electronics). A ^57^Co source was used, and the velocity scale and isomer shift δiso were calibrated with natural iron (α-Fe-foil, 25 mm thick, 99.99% purity). An assignment of the iron species was made by a comparison of the Mößbauer parameters to literature data [34,35].

### 2.7. Computational Study

Calculations were done by using the spin-polarized DFT under Kohn–Sham formalism, implemented in Quantum Espresso [36]. Projector augmented wave (PAW) was used to represent core electrons [36]. Exchange-correlation energy functional was expressed by using generalized gradient approximation by Perdew–Burke–Ernzerhoff (GGA-PBE) [37]. Plane-wave basis sets were used with energy cut-off of 400 eV. The integration on Brillouin zone is done in 4 × 4 × 1 grid. 2 type surfaces are modeled to evaluate HO_2_^−^ generating process. Two type surfaces were (a) Fe coated with carbon using graphene on 4 layers of Fe(001) and (b) graphene as a reference. Adsorption molecule, graphene and upper half layers of Fe(001) were relaxed and lower half layers of Fe(001) were fixed to evaluate the most stable structure of the adsorbed molecules. For calculations of O_2_^2^^−^ adsorption as an initial state [38], the total charge in the unit cell was −2 (two additional electrons). 

## 3. Results

**Figure 1:**
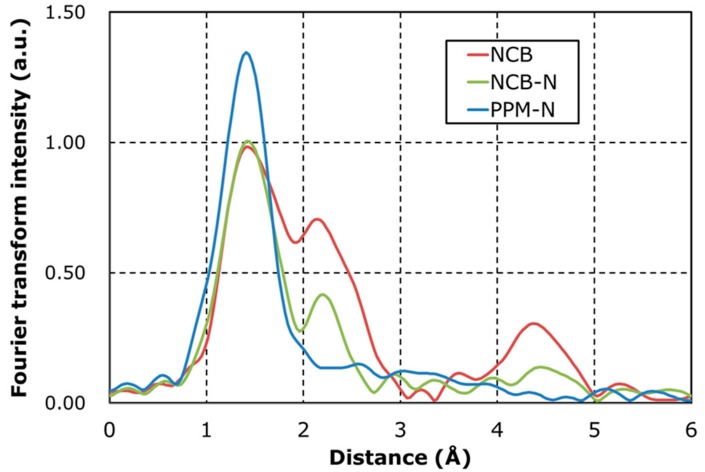
Radial structure function around Fe, calculated from the Fourier-transforms of the Fe K-edge extended X-ray absorption fine structure (EXAFS) spectra of NCB, NCB-N, and PPM-N.

Figure 1 shows Fourier-transforms of the Fe K-edge extended X-ray absorption fine structure (ex-situ EXAFS) spectra for three samples of electrocatalysts. Synthesized Fe-N-C electrocatalysts have two peaks. The first nearest neighbor peak around 1.6 Å is assigned to the Fe-N_x_ structure [39]. The second peak around 2.2 Å is assigned to Fe-Fe originating from Fe metallic particles. NCB represents the peak of Fe-Fe higher than NCB-N and PPM-N, indicating that acid treatment with HNO_3_ facilitates removal of Fe metallic particles. PPM-N represents the lowest peak of Fe-Fe and a higher peak of Fe-N_x_ rather than the others, indicating that using PIPEM as precursor reduces the amount of Fe metallic particles and increases the amount of Fe-N_x_ structures. To reveal the relationship between the structure of Fe-N-C electrocatalysts and ORR activity (Equations (2) and (3)), RRDE analysis were done.

Dual ORR reactions consist of the following two reactions:

O_2_ + H_2_O + 2e^−^ → HO_2_^−^ + OH^−^ (Ε_0_ = +0.761 V vs. RHE, pH = 14) (2)

HO_2_^−^ + H_2_O +2e^−^ → 3OH^−^ (Ε_0_ = +1.693 V vs. RHE, pH = 14) (3)

**Figure 2:**
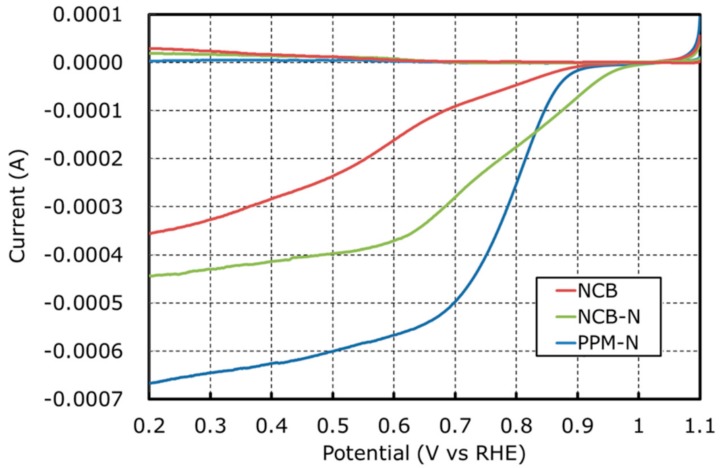
Voltammograms on NCB, NCB-N, and PPM-N of oxygen reduction reaction with rotating ring-disk electrode in 1.0 M KOH at room temperature.

**Figure 3:**
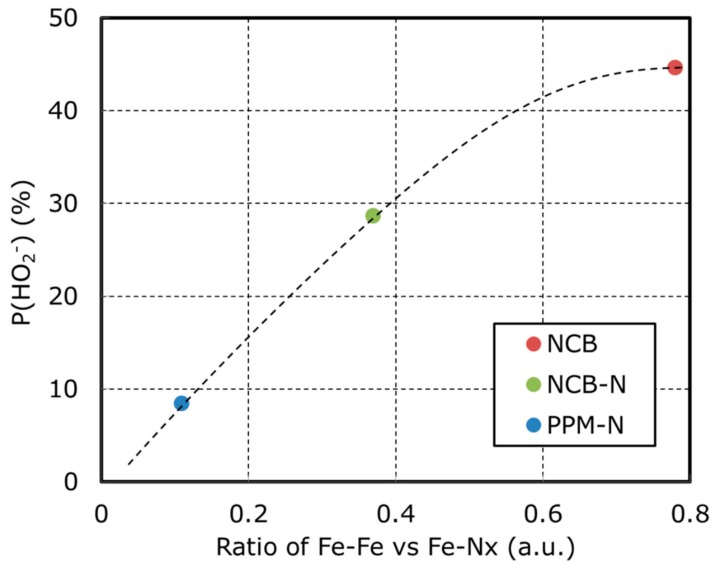
Voltammograms on NCB, NCB-N, and PPM-N of oxygen reduction reaction with rotating ring-disk electrode in 1.0 M KOH at room temperature.

Figure 2 and Table 2 show a comparison of RRDE results. NCB produces high amounts of HO_2_^−^ and results in low kinetic currents and onset/half wave potential. It suggests that the process to generate HO_2_^−^ via Equation (2) is enhanced and HO_2_^−^ reduction via Equation (3) does not progress. On the other hands, PPM-N produces low amounts of HO_2_^−^ and results in high kinetic currents and onset/half wave potential because some of the remaining Fe particles are removed by the second leach of HNO_3_. This suggests that the reduction of HO_2_^−^ via Equation (3) is more active for PPM-N than for NCB. NCB-N is in between NCB and PPM-C. To discuss the effect of Fe-N-C structures (from Figure 1 and Table 3) to HO_2_^−^ generation, the relationship between P(HO_2_^−^) and the ratio of Fe metallic particles/Fe-N_x_ is plotted in Figure 3. Figure 3 represents that P(HO_2_^−^) is in proportion to the ratio of Fe metallic particles/Fe-N_x_. From this result, it is hypothesized that Fe metallic particles enhance HO_2_^−^ generation represented by Equation (2). To confirm this hypothesis, it is necessary to discuss ORR mechanism on the surface of Fe metallic particles. STEM analysis has been done to investigate surface structure of Fe metallic particles.

**Figure 4:**
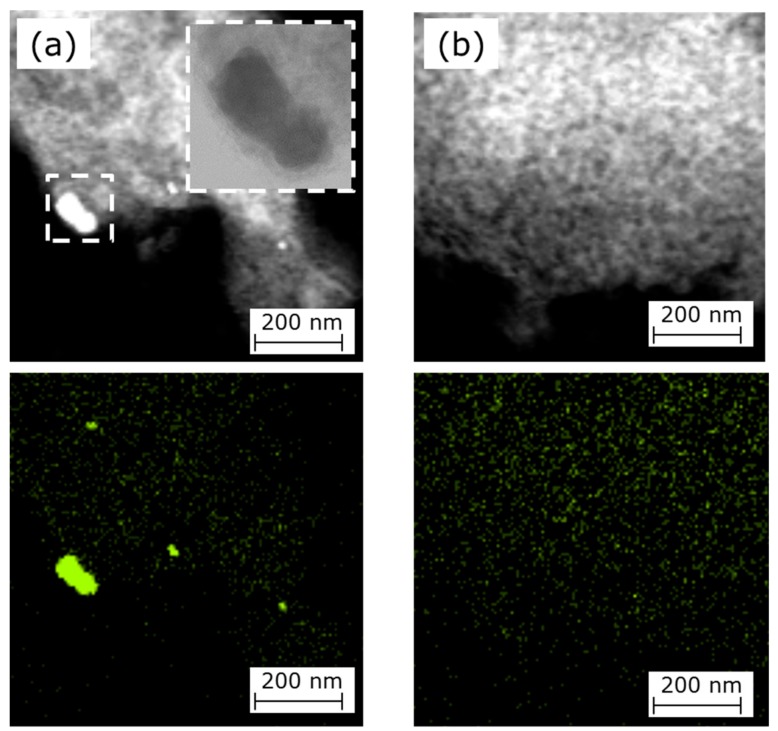
HAADF-STEM images and EDS mapping images of Fe, (**a**) NCB and (**b**) PPM-N.

Figure 4 shows HAADF-STEM images and EDS mapping images of Fe for NCB, which represents the high amount of Fe metallic particles, and PPM-C as a reference. The results presented on Figure 4 represent good agreement with the results of EXAFS in Figure 1. Aggregated Fe metallic particles from 10 to 100 nm were observed in NCB (Figure 4a). Atomically distributed Fe, which was assigned as Fe-N_x_, was observed in PPM-N (Figure 4b).

**Figure 5:**
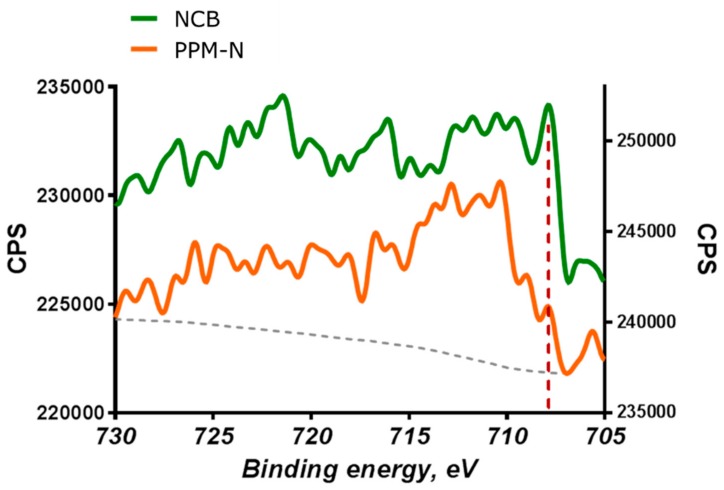
Fe2p HAXPES spectra of NCB and PPM-N. The dot line represents binding energy of metallic Fe: 707 eV.

In Figure 5, HAXPES shows the difference in amount of Fe metallic particles between NCB and PPM-N. These results also represent good agreement with the results of EXAFS in Figure 1 and HAADF-STEM in Figure 4.

**Figure 6:**
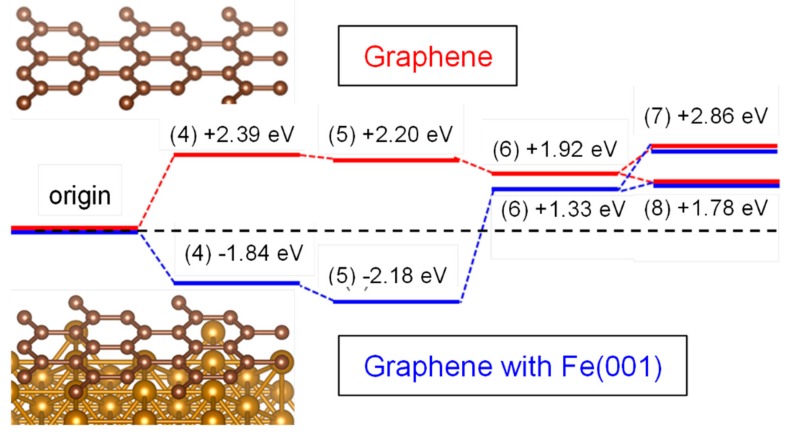
The energy diagrams for the reactions from O_2_ adsorption to generate HO_2_^−^ or OH^−^.

Additionally, Figure 4a shows that Fe metallic particles were coated with a carbon layer. It is well known that carbon in alkaline condition is a strong HO_2_^−^ generator [40]. Therefore, it is assumed that HO_2_^−^ generation by carbon is enhanced by Fe metallic particle substrate. To investigate the interaction between Fe metallic particle substrate and carbon overlay, theoretical studies (DFT calculation) were done. Figure 6 presents the energy diagrams for the reaction on carbon (graphene) with Fe substrate (Fe(001) slab [41]) and on only carbon as a reference. It is well known that the edge structure of carbon enhances HO_2_^−^ generation rather than terrace structure [42]. We assumed that the effect of Fe substrate would be present on the terrace but not on the edge. Hence DFT calculations were done based on graphene (as terrace carbon) on Fe(001) slab.

In this computational part, we focused on the calculation of the energy diagrams of the following reactions [32,39]:

O_2_ + H_2_O + 2e^−^ → *O_2_^2^^−^ + H_2_O: O_2_ adsorption (4)

*O_2_^2^^−^ +H_2_O → *(O_2_ + H_2_O)^2^^−^: H_2_O adsorption (5)

*(O_2_ + H_2_O)^2^^−^ → *HO_2_^−^ + OH^−^: HO_2_^−^ formation (6)

*HO_2_^−^ +OH^−^ → *O^−^ + 2OH^−^: HO_2_^−^ reduction (7)

*HO_2_^−^ +OH^−^ → HO_2_^−^ + OH^−^: HO_2_^−^ dissociation (8)

* represents the adsorbed X molecule on the surfaces. We compared the energy diagram on graphene with Fe(001) and graphene. By making such comparisons, we could observe the possible rate-limiting step for HO_2_^−^ generation on each surface.

In the case of graphene, the step of O_2_ adsorption (Equation (4)) required high energy as shown in Figure 6. This indicates that graphene without Fe metallic particle substrate was not so active for HO_2_^−^ or OH^−^ generation.

**Figure 7:**
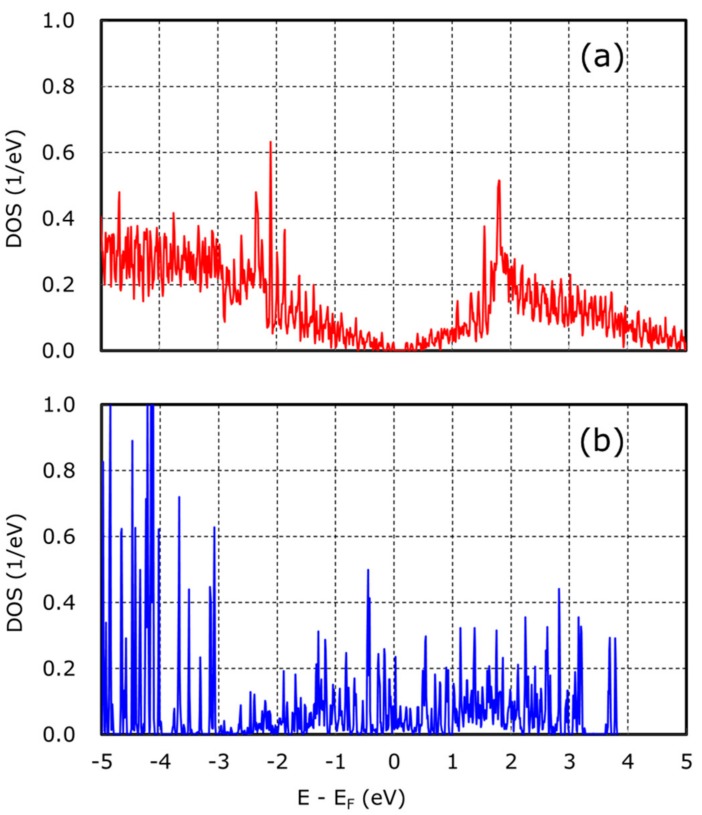
DOS of (**a**) graphene, and (**b**) graphene with Fe(001).

On the other hand, there is no energy barrier in case of graphene with Fe(001), just requiring endothermic energy to generate HO_2_^−^: +1.78 eV/OH^−^: +2.86 eV. Moreover, O_2_ and H_2_O adsorption energy: −2.18 eV is available on graphene with Fe(001). This indicates that endothermic energy required for the process from O_2_ to HO_2_^−^ is given by another O_2_ and H_2_O adsorption. From these results, it is confirmed that Fe metallic particle substrate enhanced HO_2_^−^ generation. To reveal this mechanism, the investigations of the density of state (DOS) of graphene without/with Fe(001) were done. The comparison of DOS is shown in Figure 7.

Figure 7a shows that graphene without Fe(001) had a band gap around Fermi energy, indicating semiconductive character. On the other hands, Figure 7b shows that graphene with Fe(001) had an electric state around Fermi energy, indicating conductive character. Therefore, charge transfer from graphene to adsorbed O_2_ was more favorable on graphene with Fe(001) rather than graphene without Fe(001). We conclude that this difference of DOS affected required energy for O_2_ adsorption (energy barrier for HO_2_^−^ generation).

**Figure 8:**
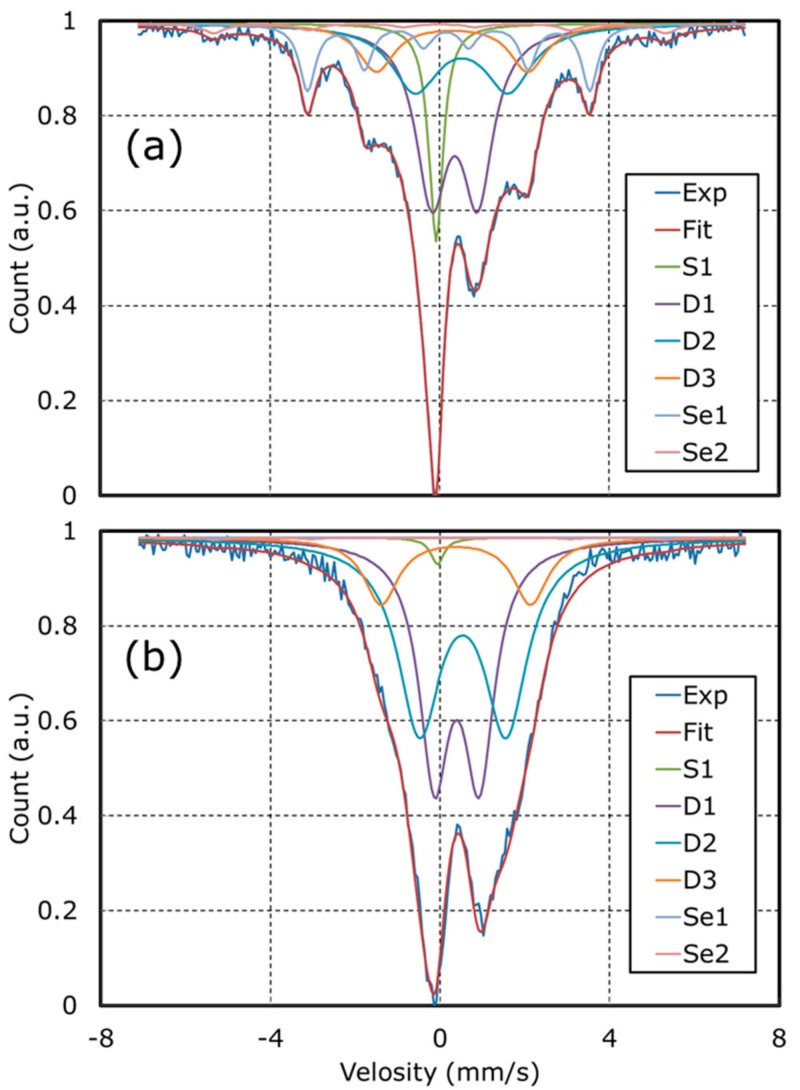
Deconvoluted Mößbauer spectra of (**a**) NCB and (**b**) PPM-N.

From the ex-situ XAFS, RRDE, STEM, HAXPES and theoretical studies, we confirmed that Fe metallic particles enhance HO_2_^−^ generation. To design reactive Fe-N-C catalysts, the role and active site of the other type of Fe structure present: Fe-N_x_ should be investigated. To identify the detail of Fe-N_x_ structures and reveal the relationship between Fe-N_x_ structure and catalytic activity, Mößbauer measurements were done. Figure 8 and Table 4 show the results.

The results of Mößbauer measurements represented good agreement with the results of EXAFS in Figure 1 and HAADF-STEM in Figure 4. The high amount of Fe metallic particles assigned γ-Fe, FeC and α-Fe were detected in NCB and high amount of Fe-N_x_ structure assigned D1, D2 and D3 sites were detected in PPM-N as shown in Table 4. Here, D1 was assigned to Fe^II^N_4_/C (low spin), D2 was assigned to Fe^II^N_4_ (like Fe-Phthalocyanine) and D3 was assigned to N-Fe^II^N_2+2_/C (high spin) sites, respectively [34,35]. The amount of D2 is in proportion to the catalytic activity (confirmed by kinetic currents, half-wave potential and onset potential in Table 2). From these results, it is hypothesized that D2 enhances ORR occur-ring through Equations (2) and (3). To confirm this hypothesis, in-situ XAFS analysis was done to investigate the change of adsorption structure in ORR.

**Figure 9:**
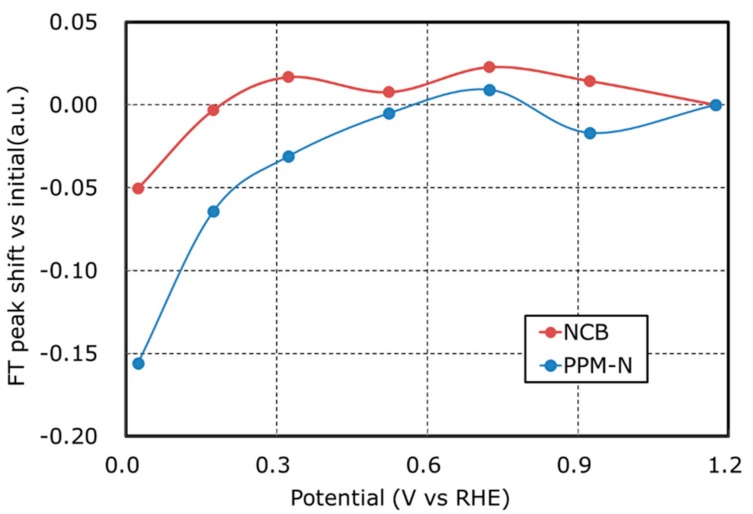
FT peak shift vs initial calculated from in-situ EXAFS data for NCB and PPM-N.

Figure 9 shows the results of in-situ analysis of EXAFS on NCB and PPM-N, respectively. To discuss the change of coordination number of Fe-N_x_, peak shift at first nearest neighbor peak of Fe around 1.6 Å is plotted in Figure 9. Figure 9 represents that the peak of PPM-N is decreased by the potential shift from 0.25 V to −0.9 V (vs. Hg/HgO). This suggests that adsorbed O_2_ at the initial state (0.25 V vs. Hg/HgO) on the surface of PPM-N is dissociated and reduced to OH^−^.

From the results of Mößbauer measurements and in-situ XAFS, we assumed that D2 enhances ORR process. However, in-situ XAFS does not directly suggest a change in the structure of D2 (including D1 and D3). For certification of this assumption, more studies (e.g., DFT calculation for ORR on D1, D2 and D3) are required in future work.

## 4. Conclusions

To reveal the mechanism of HO_2_^−^ generation from Fe-N-C catalysts, three different Fe-N-C catalysts were analyzed with ex-situ XAFS, STEM, RRDE and DFT calculations. It was revealed that carbon overlay on Fe metallic particles changes the material from a semiconductor to a conductor and enhanced HO_2_^−^ generation. 

Additionally, Mößbauer measurements and in-situ XAFS analysis were performed to better understand the structure of active sites. They suggested that a D2, assigned to Fe^II^N_4_ (like Fe-Phthalocyanine), structure enhances catalytic activity. It is expected that the research will contribute to the further development of PGM-free electrocatalysts as nanomaterials, leading to widespread popularization of environmentally friendly fuel cells.

## Figures and Tables

**Table 1:** Synthesized Fe-N-C electrocatalysts.

Catalyst (Abbreviation)	Precursor	1st Acid Treatment	2nd Acid Treatment
NCB	Nicarbazin	20 wt. % HF	-
NCB-N	Nicarbazin	20 wt. % HF	1 M HNO_3_
PPM-N	Pipemidic acid	20 wt. % HF	1 M HNO_3_

**Table 2:** Electrochemical performance of NCB, NCB-N, and PPM-N using RRDE.

Catalyst	P(HO_2_^−^) N = 0.38	I_d_ (mA) @ 0.2 V vs. RHE	Onset Potential (V) vs. RHE	Half Wave Potential (V) vs. RHE
NCB	44.6	−0.35	1.01	0.57
NCB-N	28.6	−0.44	1.04	0.74
PPM-N	8.4	−0.67	1.04	0.78

**Table 3:** Ratio of Fe-Fe/Fe-N_x_ which is calculated from EXAFS fitting.

Catalyst	Fe-N_x_ (Area)	Fe-Fe (Area)	Fe-Fe/Fe-Nx (ratio)
NCB	0.68	0.53	0.78
NCB-N	0.65	0.24	0.37
PPM-N	0.81	0.09	0.11

**Table 4:** Difference of components of the Fe-N-C electrocatalysts by Mößbauer spectroscopy.

Catalyst	S1 (γ-Fe)	Se1 (FeC)	Se2 (α-Fe)	D1	D2	D3
NCB	10.8	15.3	2.9	36.9	21.8	12.3
NCB-N	10.0	0.0	0.7	36.1	37.0	16.2
PPM-N	1.1	0.0	0.5	39.8	45.7	12.9
